# Integrated physicochemical, hormonal, and transcriptomic analysis reveals the underlying mechanism of callus formation in *Pinellia ternata* hydroponic cuttings

**DOI:** 10.3389/fpls.2023.1189499

**Published:** 2023-06-20

**Authors:** Xiaoqing Duan, Lin Chen, Youping Liu, Hongping Chen, Fu Wang, Yuan Hu

**Affiliations:** State Key Laboratory of Southwestern Chinese Medicine Resources, Department of Pharmacy, Chengdu University of Traditional Chinese Medicine, Chengdu, China

**Keywords:** *P. ternate*, histomorphological, phytohormone, transcriptome, plant hormone signaling and synthesis pathways

## Abstract

**Introduction:**

*P. ternata* is a perennial herb of the family Araceae that grows in China and has various medicinal properties and applications. At present, the artificial cultivation of *P. ternata* is constrained by seedling propagation. To address the problems of low seedling breeding propagation efficiency and high cost, our group has developed a highly efficient cultivation technology for "hydroponic cuttings of *P. ternata* "for the first time. *P. ternata* is used as the source material and is grown in a hydroponic system, increasing the seedling production rate 10-fold compared with the traditional cultivation mode. However, the callus formation mechanism in cuttings from hydroponic cultivation is still remains unclear.

**Methods:**

In order to better understand the biological process of callus formation in cuttings from hydroponic *P. ternata*, anatomical characterization, endogenous hormone content determination and transcriptome sequencing were performed on five callus stages from early growth to early senescence.

**Results:**

Regarding the four major hormones during the callus developmental stages of *P. ternata* hydroponic cuttings, cytokinins showed an increasing trend during callus formation. IAA(indole-3-acetic acid) and abscisic acid contents increased at 8d and then decreased, while jasmonic acid content gradually decreased. A total of 254137 unigenes were identified by transcriptome sequencing in five callus formation stages. Kyoto Encyclopedia of Genes and Genomes (KEGG) enrichment analysis of the differentially expressed genes (DEGs) that differentially expressed unigenes were involved in various plant hormone signaling and hormone synthesis-related pathways. The expression patterns of 7 genes were validated using quantitative real-time PCR.

**Discussion:**

This study presented integrated transcriptomic and metabolic analysis approach to obtain insights into the underlying biosynthetic mechanisms and function of key hormones involved in the callus formation process from hydroponic *P. ternata* cuttings.

## 1 Introduction

The medicinal plant *P. ternata* is widely used in China, Korea, and Japan (Bai et al., 2022) and has been proven to be very effective in treating cough, vomiting, infections, and inflammatory diseases (Peng et al., 2019; Peng et al., 2022). Modern pharmacological studies have demonstrated that it possesses a variety of activities such as cough suppressant, expectorant, anti-vomiting, anti-tumor, anti-bacterial, hypnotic, and others (Kong et al., 2018). It is used in various aspects of Chinese medicine, including clinical formulations of Chinese medicine and raw materials of many types of patented Chinese medicines, among which more than 400 Chinese patented medicines such as Huoxiang Zhengqi Oral Liquid, Banxia Cough Tablets and Banxia Tianma Pills (Mao and He, 2020). The current market demand for *P. ternata* exceeds 8,000 tons annually, far exceeding the sum of the current cultivated and wild *P. ternata* production. The disparity between supply and demand is outstanding (Kumari et al., 2017). The shrinkage of wild resources and the low degree of mechanization seriously restrict the increase of production. The cultivation and management techniques are not in place, resulting in low yield and uneven quality of the herb, which seriously affects the quality and clinical efficacy of *P. ternata*.

Currently, the *P. ternata* market is in a state of oversupply. Appropriate propagation methods, including seeds, tubers, bulbil, and group culture, have been evaluated domestically and abroad. Seed propagation faces problems such as low reproduction coefficient, long reproduction cycle, and quality degradation. As the medicinal part of *P. ternata* is the tuber, there is a contradiction between seed retention and the commercial use of tuber propagation. Bulbil propagation generally takes 2 to 3 years before the bulbils are used for medicinal purposes, and there are issues with the long propagation cycle. Rapid propagation of *P. ternata* using seedlings in tissue culture has been reported under laboratory conditions. This method requires steps such as sowing, rooting, and transplanting, which are demanding and complicated. Therefore, there is an urgent need to explore more convenient breeding and propagation methods to expand the propagation resources of *P. ternata.*

To expand the *P. ternata* resources and explore a more convenient breeding method, our group has established a system of cuttings obtained from the hydroponic culture of *P. ternata*, in which we induced the formation of callus on the cut surface of leafy stems above the Bulbils of *P. ternata*. These were further differentiated to form multiple adventitious buds without using exogenous hormones and further developed and matured to form tubers. Plants exhibit multiple regeneration patterns after trauma to a body part (Birnbaum and Alvarado, 2008; Ikeuchi et al., 2016). Plant regeneration is characterized by the formation of new shoots and/or roots through organogenesis (*de novo* organogenesis) from plant tissues. Callus formation is the protective response after a plant is injured and regenerates new organs. The wound-induced callus is formed at the wound site, preventing water loss and serving as a source of cells for vascular differentiation and/or *ab initio* organogenesis (Ikeuchi et al., 2013; Ikeuchi et al., 2016). Cells near the wound proliferate and establish new shoots or root tip meristematic tissues, giving rise to new organs. These newly formed meristematic tissues can be derived directly from the parent plant or indirectly from the callus formed at the wound (Ikeuchi et al., 2013). Many plants can regenerate from shoots or root explants, and this property has contributed to the increased possibility of asexual reproduction of stem segments. Surprisingly, in some plant species, whole plants can develop through certain plant tissue regeneration (Nakayama et al., 2014). Currently, only a few species can regenerate whole plants when the cut damage occurs naturally. Still, in this study, we found that after cutting the stem, the stem segment of *P. ternata* can regenerate to form a complete plant in pure water cutting culture. Callus is a key link in the development and maturation of hydroponic cuttings of *P. ternata* to form tubers, so exploring the mechanism of callus formation is currently a key priority.

Callus proliferation and differentiation are regulated by the expression of multiple genes (Chu et al., 2017; Ferdausi et al., 2021). The growth of the model plant *Arabidopsis thaliana* is regulated by various genes in an auxin-rich media, such as the *PLT* family of lateral root regulators, the *LBD* family of root formation regulators (Fan et al., 2012), the *WOX5* root meristem regulator genes (Sugimoto et al., 2010), and *WOX11-LBD16*, which promotes the callus totipotency acquisition. Moreover, several *APETALA2*/*ethylene response factor* (*AP2/ERF*) transcription factors termed *trauma-induced dedifferentiation 1* (*WIND1*), *WIND2*, *WIND3*, and *WIND4* play key roles in trauma-induced callus formation (Iwase et al., 2011). Recent studies have shown that *WIND1* directly upregulates the expression of the Arabidopsis *AP2/ERF* transcription factor *ENHANCER of SHOOT REGENERATION1* (*ESR1*) to promote callus formation and plant regeneration (Iwase et al., 2017). Auxin can stabilize the expression of *AtbZIP59*, and in *AtbZIP59* mutants, auxin can maintain callus formation, while overexpression of *AtbZIP59* can promote callus formation (Xu et al., 2018). In addition, *MYB94* and *MYB96* can directly control *AtLBD29* expression, and plants overexpressing *AtLBD29* showed significant phenotypic changes during growth and development, inhibiting callus inhibition (Dai et al., 2020). JA is a trauma-induced hormone (Creelman et al., 1992) and has a slight inhibitory effect on callus formation at wound sites (Ikeuchi et al., 2017). ABA transduces various stress responses, such as drought and heat stress, but only a few studies have reported its involvement in trauma-induced signaling (Pēna-Cortés et al., 1989; Pena-Cortes et al., 1991; Dammann et al., 1997). Cytokinins (CKs) are a large group of plant hormone promoting cell division and prevent senescence. Post-trauma–induced CKs responses have been found to regulate callus formation (Ikeuchi et al., 2017). IAA plays a key role in regulating root regeneration after injury (Chen et al., 2016), and it also promotes the formation of callus in tissue culture. Although the molecular mechanisms of callus differentiation have been reported in many plant species, the genes involved in the proliferation and differentiation of *P. ternata* callus have not yet been adequately studied, and the underlying molecular mechanisms are still unclear.

In order to elucidate the molecular mechanism underlying of the hydroponic cutting callus formation in *P. ternata*. Through analysis of the histological characteristics and hormonal metabolism of callus samples at different stages, we identified changes in four major classes of plant hormones during callus formation in P. ternata hydroponic cuttings. Our findings showed that the total CK and auxin contents are crucial for callus formation, highlighting their importance during callus formation. We also analyzed the transcriptome dynamics of five stages of *P. ternata* callus development and examined the expression of genes involved in phytohormone biosynthesis and signaling pathways. Our study revealed variations in gene expression at different callus stages, suggesting that callus formation is a complex process that involves multiple genes and interconnected mechanisms. This study investigated the molecular mechanism of callus formation in *P. ternata* hydroponic cuttings, laying the groundwork for further investigation of the molecular regulatory network underlying callus formation and providing a theoretical basis for expanding *P. ternata* resources.

## 2 Materials and methods

### 2.1 Plant material and experiment at conditions

*P. ternata*, originally harvested from Qingshan Village, Yingshan County, Nanchong City, Sichuan Province, was collected from the Chengdu University of Traditional Chinese Medicine. P. ternata were planted in small potting trials in river sand with humidity maintained at 85% and temperature at 28°C. When the bulbils were mature (brown), the three-leave stem above the bulbils were collected as cuttings. For disinfection, the cuttings were washed with sterile water. Afterward, the cuttings were inserted into a sterilized foam plate and immediately placed into a hydroponic tank. The basal cut of the stem cutting was immersed in 0.1 mg/liter of 6-BA (6-benzyladenine) solution for culture. The concentration of the 6-BA solution was determined according to agricultural practices. The culture was incubated in an air-conditioned room at 28°C, 14/10h light/dark and 75% relative humidity. The 6-BA culture solution was changed once a week.

At 0, 8, 15, 25, and 30 days, 120 stem cuttings from each period (40 samples per replicate) were collected from the base of stem segments (approximately 2.0 cm of callus growth). A part of the callus was preserved in 4% paraformaldehyde for histological morphological observation by staining with Saffron solid green (SSG) and Periodic acid-Schiff (PAS). The other sampled callus tissue was snap-frozen in liquid nitrogen and stored at −80°C in an ultra-low temperature refrigerator for determination of oxidase activity, transcriptome analysis, and targeted endogenous hormone content determination.

### 2.2 Histomorphological, histochemical, and biochemical characterization

Fresh callus was photographed under a camera for observation. Light microscopic observation was performed using cross-sectional dissection after fixation of the callus in 4% paraformaldehyde, and paraffin sections were embedded. Sections (5 μm in thickness) were cut using a Leica RM2235 (Leica, Shanghai, China) rotary sectioning machine. Cell proliferation was observed using staining with SSG. Insoluble polysaccharides and starch were detected by staining with PAS.

### 2.3 Analysis of peroxidase, phenylene oxide, and indoleacetic acid oxidase

The enzymatic activities of peroxidase (POD), polyphenol oxidase (PPO), and indoleacetic acid oxidase (IAAO) were assessed in the callus samples according to the method used by Zhang et al^1^. The sample (0.5 g) was ground into a homogenate in 1 ml of cold phosphate buffer (50 mM, pH 6.5) and a small amount of quartz sand in a mortar in an ice bath. After diluting the homogenate to 2 ml, the supernatant was collected by centrifugation (1790 × g, 20 min, 4°C) and readjusted to a constant volume. For analysis of IAAO, 1 ml of enzyme solution was added to the reaction solution (1 ml 1×10^−3^ MnCl_2_, 1 ml 1×10^−3^ M 2,4-dichlorophenol, 2 ml 1×10^−3^ M IAA (indole-3-acetic acid) and 5 ml of phosphate buffer and mixed in tube 1. The same volume of phosphate buffer was added to the same reaction system in tube 2. Distilled water was added to tube 3 as a control. Then, 1 ml of each mixed solution was added to the addition of 2 ml of FeCl_3_ perchlorate reagent, shaken, and incubated for 30 min at 30°C in the dark. Absorbance values were measured at 530 nm. For analysis of PPO, after a 1-min reaction in 2.5 ml of phosphate buffer (pH 6.0) 0.5 ml enzyme solution, 1 ml catechol (10 M) was added and shaken. The absorbance was measured at 420 nm. For POD analysis, 0.1 ml of enzyme solution was added to the reaction solution, 0.1 ml of enzyme solution was added to the reaction solution (2.9 ml of 0.05 M phosphate buffer, 1.0 ml of 2% H_2_O_2_, 1.0 ml of 0.05 M guaiacol) and incubated in a water bath at 30°C for 3 min. Then, the absorbance values were measured at 470 nm.

### 2.4 Plant hormone determination

When the cuttings were obtained, there was no callus formation, and the 2 cm of tissue at the base of the stem was used for the phytohormones determination. Afterward, when the callus was formed, the callus’s phytohormone content at the stem’s base was measured in five time periods. The harvested callus from the five-time periods was immediately frozen in liquid nitrogen, ground into powder (30 Hz, 1 min) using a ball mill (MM400, Retsch, Germany), and stored at −80°C until needed. 50 mg of plant sample was weighed into a 2 ml of plastic microtube and frozen in liquid nitrogen, dissolved in 1 ml of methanol/water/formic acid (15:4:1, V/V/V). Ten microliter internal standard (IS) mixed solution (100 ng/ml) was added into the extract as IS for the quantification. The mixture was vortexed for 10 min, then centrifugation for 5 min (12000 r/min and 4°C), the supernatant was transferred to clean plastic microtubes, followed by evaporation to dryness and dissolved in 100 μl of 80% methanol (V/V), and filtered through a 0.22-μm membrane filter for further LC-MS/MS analysis (Floková et al., 2014; Li et al., 2016). Phytohormones contents were determined by MetWare^2^based on the AB Sciex QTRP6500+LC-MS/MS platform. All assays were performed with three biological replicates.

### 2.5 RNA extraction, library preparation, and RNA-seq

Total RNA was extracted using the Plant RNA Extraction Kit (Tiangen, Beijing, China). RNA concentration and integrity were assessed using a Qubit 2.0 fluorometer (Life Technologies, California, USA) and an Agilent 2100 Bioanalyzer (Agilent Technologies, California, USA). Each sample (≥1 μg) was used for sequencing library preparation using the NEBNext® Ultra™ RNA Library Preparation Kit (NEB, USA) according to the manufacturer’s instructions. Library quality was subsequently assessed by an Agilent 2100 Bioanalyzer system. The Illumina NovaSeq 6000 platform was used for subsequent sequencing.

### 2.6 Data filtering and transcriptome analysis

After removing adapter sequences, ambiguous reads, and low-quality reads, the remaining high-quality clean reads were used for subsequent analysis. Transcript assembly was performed on clean reads using Trinity, and the transcripts obtained from the assembly were clustered and de-redundant using Corset^3^. ClusterProfile software was used for GO (Gene Ontology) functional analysis, and the KEGG (Kyoto Encyclopedia of Genes and Genomes)^4^ database was used for pathway enrichment analysis. OmicShare tool^5^ was used to graphically display the results of GO and KEGG functional enrichment analysis with adjusted *p* values.

### 2.7 Identification of differentially expressed genes

Fragment per kilobase fragment per million fragment transcripts (FPKM) values were used to normalize gene expression. The DESeq2 R software (1.16.1) was used to identify differentially expressed genes (DEGs, 0d vs. 8d, 15d, 25d, and 30d) according to the following threshold criteria:|log_2_ (fold change) |>1, *p* adjusted < 0.05. Pearson correlation coefficients were used to assess correlation of the biological replicates.

### 2.8 Quantitative real-time polymerase chain reaction assay

For expression validation of differentially expressed genes, special primers were designed for seven differentially expressed genes were selected by using Premier 5.0 software. The specificity of each primer set was assessed with polymerase chain reaction (PCR) amplicon sequencing, and the efficiencies of the different primer sets were similar. Details of all primers are described in **Supplementary Table S9**. Total RNA, initially treated with DNase I Amplification Grade enzyme (Tsingke, Beijing, China), was used as the template for the reverse transcriptase reactions. An aliquot of treated RNA was used in quantitative polymerase chain reaction (qPCR) to rule out DNA contamination. Complementary DNA (cDNA) synthesis was done using Super Script First-Strand Synthesis System for RT-PCR (Tsingke, Beijing, China) with random hexamers and oligo (dT) primers. qPCR reaction was performed in a volume of 20 μl containing 10 μl 2 × All-in- One TM (Gene Copoeia, Los Angeles, USA) qPCR Mix, 2 μl of cDNA, 1 μl of each 4 μM primer, and 6 μl RNase-free sterile water. To normalize the relative expression of selected genes, the GAPDH gene was used as a reference (Zhu et al., 2013). qPCR was performed using an iQ5 Real-Time PCR Detection System (Bio-Rad, Hercules, USA). PCR reactions were performed at 95°C for 2 min, followed by 40 cycles of 95°C for 15 s, 60°C for 20 s, and 72°C for 20 s. Melting curve analysis was conducted for each reaction to confirm the specificity of the reaction, and all the cDNA samples were analyzed in triplicate. The relative expression levels of candidate genes were calculated using the 2−ΔΔCt method.

### 2.9 Statistical analysis

Statistical processing of plant hormone content and qRT-PCR data was performed in Excel. The experiments were conducted in triplicate, and data were analyzed using standard analysis of variance (ANOVA) followed by Duncan’s multiple range test (DMRT) through Origin software.

## 3 Results

### 3.1 Morphological and histological analysis

**Figure 1:**
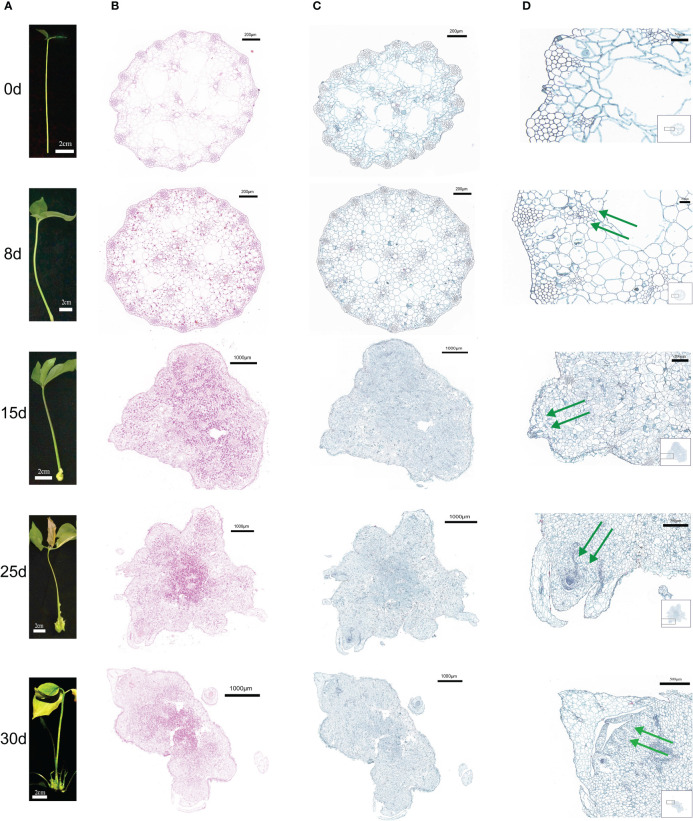
Morphological and histological characteristics of callus from *P. ternata* hydroponic cuttings cultured in water at different induction stages (0d, 8d, 15d, 25d, and 30d). **(A)** External morphology of different callus in order from top to bottom. **(B)** PAS staining of transverse callus sections at different points in order from top to bottom. **(C)** Staining of callus cross sections at different periods of time in order from top to bottom. **(D)** Local organization of saffron green in cross sections of callus at different induction stages periods of time in order from top to bottom, with green arrows indicating the process of bud formation at different induction stages. The scale bars are 2 cm, 200 μm, 1000 μm, and 500 μm, in that order.

The first morphological change in the process of callus formation occurred in the first eight days of culture(8d), when the tissue at the base of the cuttings expanded slightly and strong cell proliferation was observed on its surface. Callus cell were produced that were larger in size and grew more vigorously than those in the control group(0d) (**Figure 1**). The callus continued to grow, and after 15 days of culture (15d), instead of the initially loosely arranged cells, the callus cells at the outermost layer became relatively small and showed a high capacity to divide, thus enabling the outer layer expansion. The cells at the inner callus layers were uniformly colored and arranged without cell gaps. The callus continued to differentiate and formed shoot primordia (**Figures 1A, C**). After 25 days of culture (25d), the shoot primordium cells continued to divide, resulting in a significant increase in the number of cells and the formation of one or more shoots. After thirty days of culture (30d), multiple shoots gradually differentiated and formed stems and leaves. Histological callus observation was carried out at different time periods using a light microscope. Starch reserves were observed by staining with a periodic acid Schiff (PAS) reagent. As the time and callus growth progressed, starch accumulated in the callus, indicating that the cells were metabolically competent.

### 3.2 Variations in oxidative enzyme activity during callus formation

The callus POD, polyphenol oxidase (PPO), and indoleacetic acid oxidase (IAAO) activities at five time points of callus growth are shown in Figure (**Supplementary Figure S1**). The POD and PPO activities consistently increased, reaching their highest values on the 25th day after explant cutting (**Supplementary Figures S1A, B**). The increase in POD activity may be a result of the damage to the cuttings during excision. POD activity remained high throughout the callus formation process, probably due to the effects of exogenous hormones, that promoted the growth and development of the callus. PPO affects plant growth mainly by catalyzing the formation of IAA-phenolic acid complex. It increased rapidly during callus formation and differentiation to form buds in the early stage, producing high concentrations of the IAA-phenolic acid compounds to promote callus growth, differentiation, and bud formation. The high IAA-phenolic acid compound concentration is produced to promote callus growth and buds’ differentiation. However, after the plant is damaged or during virus infestation, PPO catalyzes the oxidation of phenols and O_2_ to form quinone, resulting in tissue browning restoring the damage, and preventing infection. In the early stage of cuttings, IAAO activity is relatively low, facilitating callus formation. At the later stage of *P. ternata* callus development, IAAO activity increased and resulted in a reduction in endogenous IAA levels, which was favorable to the differentiation of callus and the formation of shoot primordia (**Supplementary Figure S1B**).

### 3.3 Targeted metabolomic analysis of callus formation

A total of 73 metabolites of indole-3-acetic acid (IAA), CK, jasmonic acid (JA), abscisic acid (ABA), and their intermediates were measured by liquid chromatography–mass spectrometry (LC-MS) used for targeted metabolome determination at 0d, 8d, 15d, 25d, and 30d of callus formation. The results revealed 55 hormone metabolites, including 20 auxin analogs and their derivative. PCA was performed, with the first principal component explaining 48.78% of the variation and the second principal component explaining 21.58% of the variation (**Supplementary Figure S2A**). There was a clear separation between the samples of different time points in these two dimensions. The clustering of QC samples also indicated that the experimental results were reliable. All samples were within the 95% confidence interval, indicating significant differences in metabolites in different periods of callus formation in *P. ternata* hydroponic cuttings.

#### 3.3.1 Analysis of differential metabolites during the different callus formation stages

In order to evaluate metabolite changes between the different *P. ternata* callus stages, callus samples at 0d, 8d, 15d, 25d, and 30d were assessed. The OPLA-DA model was used to test for differences to eliminate the interference of irrelevant variables. Metabolites with fold change ≥ 2 and fold change ≤ 0.5 in the control (0d) and experiment groups (8d, 15d, 25d, and 30d) were selected were considered significantly different (**Supplementary Figure S2**). Compared with 0d (CK), 44, 44, 38, and 14 metabolites were differentially accumulated in 8d, 15d, 25d, and 30d callus (**Supplementary Figures S2B, C**), and a total of 29 metabolites were differed in the four comparison groups, mainly corresponding to auxin, CK, and JA metabolites (**Supplementary Figure S2D**; **Supplementary Table S1**).

#### 3.3.2 Variations in phytohormone contents during the callus formation process

##### 3.3.2.1 Cytokinins

CKs are a class of adenine derivatives. Natural CKs are divided into free-state CKs and bound-state CKs (Záveská Drábková et al., 2021). The natural free-state CKs in plants are zeatin (ZT), zeatin riboside (ZR), and dihydro zeatin (DZ), dihydrozeatin ribonucleoside (DHZR), and N6-isopentenyladenine (IP). The bound state CKs are methionyl zeatin, methionyl isopentenyl adenosine, and 3-Indolepropionic acid (iPA).

**Figure 2:**
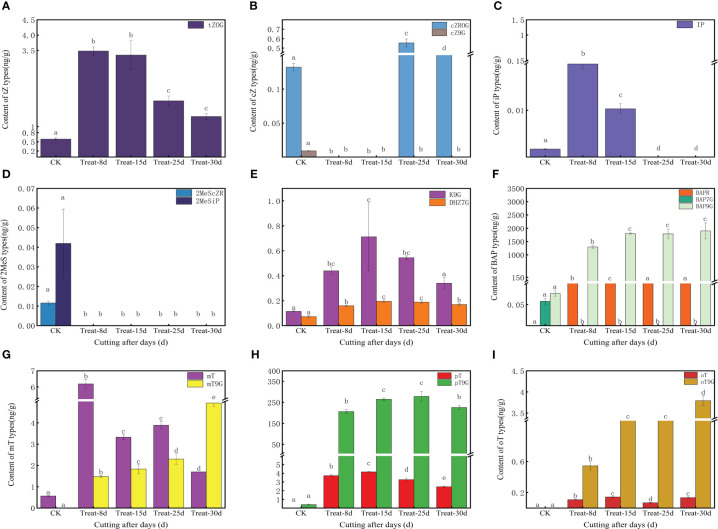
Changes in endogenous cytokinins (CKs) content at five stages of callus formation from hydroponic cuttings. **(A)** trans-zeatin (tZ) type; **(B)** cis-zeatin (cZ) type; **(C)** N6-(Δ2-prenyl) adenine (iP) type; **(D)** methyl mercaptan type (2MeScZR, 2MeSiP); **(E)** dihydrozeatin; **(F)** 6- benzylaminopurine (BAP) type; **(G)** 3-[(9H-purin-6-ylamino) methyl] phenol (mT) type; **(H)** 2-[(9H-purin-6-ylamino) methyl] phenol (pT); **(I)** 4-[(9H-purin-6-ylamino) methyl] phenol (oT) type. The bars with different letters are significantly different (*p* < 0.05). Values are means of three replicates ± SE.

The content of various forms of CKs varied in the callus during the different time periods. The tZOG (trans-Zeatin-O-glucoside) was initially significantly increased and then significantly decreased (*P* < 0.05), with the highest level measured at 8d and 15d, indicating that tZOG plays a vital role in the early callus formation stages (**Figure 2A**). The concentration decreased in the later period of differentiation during shoot formation indicating that the level of cZROG (cis-Zeatin-O-glucoside riboside) can promote the formation of shoots in the late differentiation of callus (**Figure 2A**). Both IP7G (N6-Isopentenyl-adenine-7-glucoside) and IP9G (N6-Isopentenyl-adenine-7-glucoside) in IP showed an initial increasing trend, and then they both decreased. The level of IP7G increased sharply at 15d, indicating a role in callus formation. However, IP9G showed high levels at 8d but decreased after that time point (**Supplementary Figure S3B**). Moreover, 2MeScZR (2-Methylthio-cis-zeatin riboside) and 2MeSiP (2-Methylthio-N6-isopentenyladenine) were not detected at 8d (**Figure 2D**), suggesting they do not play a significant role in callus formation process. K9G content reached its highest levels at 15d (**Figure 2E**). BAP9G (N6-Benzyladenine -9-glucoside) levels also remained high after an initial increase at 8d (**Figure 2F**). mT (meta-Topolin) decreased gradually after a significant increase at 8d, while mT9G (meta-Topolin-9-glucoside) gradually increasing (**Figure 2G**). oT9G (ortho-Topolin-9-glucoside) also showed the same trend (**Figure 2I**).

While 17 CKs were identified in the shared differentially accumulated metabolite, the clustering heat map results indicated that cZ9G, BAP9G, 2MeScZR, and 2MesiP were present in high levels only in control at 0d (**Supplementary Figure S2D**). A significant elevation of IP and mT at 8d, suggesting a potentially important role in triggering callus induction. BAPR was significant expression elevated at 15d, suggesting a role in callus growth and expansion, while cZROG was elevated at 25d, and mT9G and oT9G were elevated at 30d (**Supplementary Figure S3**).

##### 3.3.2.2 Auxin

**Figure 3:**
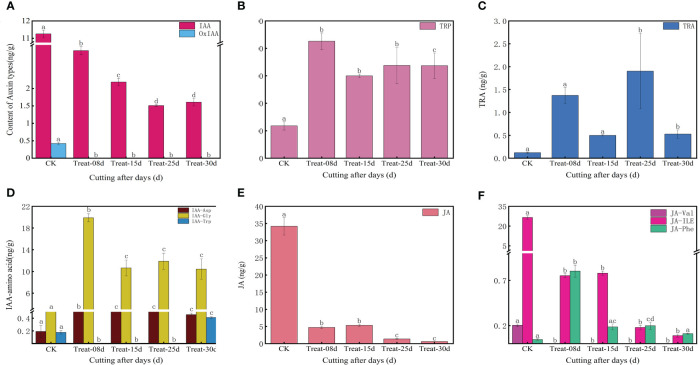
Changes in endogenous auxin, jasmonic acid, and abscisic acid contents in five stages of callus formation in *P. ternata* hydroponic cuttings. **(A)** IAA active compound; **(B)** tryptophan, a key compound for IAA synthesis; **(C)** tryptamine; **(D)** amino acid coupling substance of IAA; **(E)** JA; **(F)** amino acid conjugates of JA. The bars with different letters are significantly different from each treatment (*p* < 0.05). Values are means of three replicates ± SE.

The endogenous auxin content changed significantly during the formation of callus in *P. ternata*. The level of IAA (Indole-3-acetic acid) decreased significantly (*P* < 0.05) from the cuttings excision date and remained low during the formation of the callus (**Figure 3A**). The level of MEIAA (methyl indole-3-acetate) increased significantly (*P* < 0.05) at 8d and remained elevated, indicating that MEIAA was beneficial for callus induction (**Supplementary Figure S4A**). IBA (3-Indolebutyric acid) is another form of auxin in plants, which is more stable than IAA, but its levels were low in this experiment (**Supplementary Figure S4A**). IAN (indole-3-acetonitrile) and IAM (indole-3-acetamide) act as important precursors for IAA synthesis and determine the amount of IAA synthesized. The level of IAM increased sharply at 8d (*P* < 0.05) during callus. Still, its level was 0 at 15d, 25d, and 30d, indicating that IAM facilitates callus initiation (**Supplementary Figure S4C**). In contrast, the content of IAN decreased at 8d and was maintained at these levels at 15d but increased again at 25d and eventually decreased at 30d (*P* < 0.05) (**Supplementary Figure S4D**). This indicates that IAN carries an important role in the late differentiation callus and in shoots formation. IAA synthesis includes both tryptophan-dependent and tryptophan-independent pathways. TRP (tryptophan) is rate-limiting for auxin biosynthesis and its derivatives. TRP content increased significantly (*P* < 0.05) on the date of cutting excision and remained high (**Figure 3B**), indicating that TRP is beneficial for callus formation. TRA (Tryptamine) is a TRP catabolism product, a precursor of indole-3-glyoxime by the action of *YUCC* enzyme, and of IAN synthesis catalyzed by indole-3-glyoxime dehydratase. In this study, the TRA content exhibited significant differences at 8d and 25d (*P* < 0.05), which are critical periods for callus formation (**Figure 3C**), indicating that TRA could positively affect the process. During auxin degradation, intermediates are formed with amino acids, which are storage forms of IAA and can have direct signaling functions. Among them, the level of amino acid conjugate of IAA-Asp (Indole-3-acetyl-L-aspartic acid) was significantly higher at 8d (*P* < 0.05), suggesting that its accumulation facilitates the initial callus formation process (**Figure 3D**).

Based on the cluster heat map of the shared differentially accumulated metabolites at different periods (**Supplementary Figure S2D**), the levels of IAA and OxIAA (2-oxindole-3-acetic acid) were highest at 0d. They decreased sharply during callus formation, while MEIAA, IAA-Gly (Indole-3-acetyl glycine), and IAA-Asp levels were highest at 8d and TRA at 25d, and IAA-Trp (Indole-3-acetyl-L-tryptophan) accumulated significantly at 30d, suggesting that the different auxin types may have different mechanisms of action at different stages of callus formation (**Supplementary Figure S4**).

##### 3.3.2.3 Stress hormones (jasmonic acid and abscisic acid)

JA (**Figure 3E**) and jasmonic acid isoleucine content (**Figure 3F**) showed a gradual decline (*P* < 0.05) during the callus formation. This indicates that JA and jasmonic acid isoleucine may not be directly involved in the process. In contrast, OPC-4 (3-oxo-2-(2-(Z)-Pentenyl) cyclopentane-1-butyric acid) levels were elevated at 8d and maintained at high levels at 15d. However, its levels were not detected at 25d and 30d, suggesting that OPC-4 plays a role in the developmental process of callus expansion. ABA was elevated at 8d and later returned to its initial level, suggesting a role for ABA in the early stages of callus formation at 8d (**Supplementary Figure S4D**). Among the shared differentially accumulated metabolites, only JA-Phe (N-[(-)-Jasmonoyl]-(l)-phenalanine) showed show significant differences at 8d (**Figure 3F**).

### 3.4 Transcriptomic analysis of callus formation

#### 3.4.1 Transcriptome sequencing quality testing, assembly, and differentially expressed gene analysis

Five callus formation stages at 0d, 8d, 15d, 25d, and 30d were selected for transcriptome sequencing. The reads were filtered and used for *de novo* transcriptome assembly in the absence of a reference genome. A total of 121.74 Gb Clean Data were obtained, with approximately 5 Gb of clean data per sample and a Q30 bases percentage greater than 91%. The 15 samples had GC content between 53.7% and 54.82%. The results, indicating that the nucleic acid sequence results had met the requirements for subsequent quality assessment of the analysis (**Supplementary Table S2**).

A total of 132,435 (52.11%) unigenes were matched to the Nr database by BLAST analysis, while 111,436 (43.85%), 98,381 (38.71%), 78766 (30.99%), and 92,351 (36.34%) (**Supplementary Table S3**). Also, a high percentage of annotated sequences in *P. ternata* callus were highly similar to *Elaeis guineensis* (16.03%) and *Phoenix dactylifera* (13.95%) (**Supplementary Figure S5**).

The raw data were analyzed using the negative binomial distribution using DESeq2^6^ software. The screening criteria were *P*-value < 0.05, |log_2_ FC| ≥ 1, and genes differentially expressed between the two groups were screened. 12,743 DEGs were upregulated, and 12,589 DEGs were downregulated in CK at 0d compared with the callus at 8d. In CK compared with the callus at 15d, 25,150 genes were differentially expressed, with 12,862 upregulated and 12,288 downregulated transcripts were detected. When comparing the CK *versus* callus at 25d, 27,509 differentially expressed, 12,671 upregulated and 14,838 downregulated transcripts were identified. In the CK *versus* the callus at 30d comparison, 24,332 differentially expressed genes, 12,397 upregulated and 11,935 downregulated transcripts were identified (**Supplementary Figure S6**). Meanwhile, a total of 11,067 co-expressed genes were detected in the four comparison groups, of which 4,309 were co-upregulated and 6,702 co-downregulated (**Supplementary Figures S6A, B**).

To visualize the DEGs in metabolic pathways, we classified these DEGs according to KEGG pathway enrichment analysis. The DEGs in the CK *versus* 8d callus comparison were mainly associated with metabolic pathways (3,978), biosynthesis of secondary metabolites (2,181), plant-pathogen interactions (596), and phytohormone signaling (521) (**Supplementary Table S5**). DEGs in the CK *versus* 15d callus were mainly associated with metabolic pathways (3,861), biosynthesis of secondary metabolites (2,145), plant-pathogen interactions (595), and plant hormone signaling transduction (558) (**Supplementary Table S6**). DEGs in the CK *versus* 25d callus were mainly associated with metabolic pathways (4,103), biosynthesis of secondary metabolites (2,246), ribosomes (909), and plant hormone signaling transduction (557) (**Supplementary Table S7**). Finally, the DEGs in the CK *versus* 30d callus comparison were mainly enriched in metabolic pathways (3,558), secondary metabolite biosynthesis (2,013), plant-pathogen interactions (575), and plant hormone signaling transduction (551) (**Supplementary Table S8**). In this study, we focused on signaling pathways such as “plant hormone signaling transduction” and metabolic pathways related to hormone biosynthesis.

#### 3.4.2 Analysis of key differentially expressed genes involved in the plant hormone signaling transduction pathways

**Figure 4:**
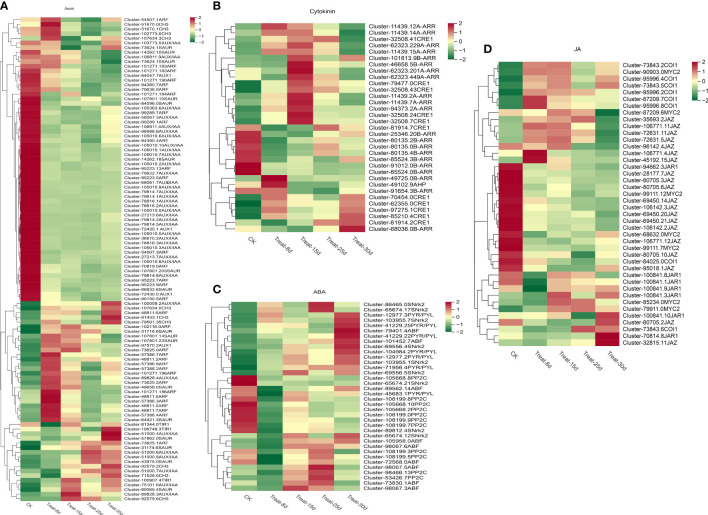
Changes in the expression levels of differentially expressed genes (DEGs) involved in auxin, cytokinin, jasmonic acid, and abscisic acid signaling pathways during the five stages of *P. ternata* callus formation. **(A)** Expression levels of DEGs in the auxin signaling pathway. **(B)** Expression levels of DEGs involved in cytokinin signaling. **(C)** Expression levels of DEGs of the abscisic acid signaling pathway. **(D)** Expression levels of DEGs of the jasmonic acid signaling pathway.

A clustering heat map was used to illustrate changes in the expression of genes associated with plant hormone signaling transduction pathways and to classify genes with similar expression patterns (**Figure 4**). A total of 253 shared DEGSs were identified as hormone related during callus development in *P. ternata*, including DEGs of auxin, CK, JA, and ABA signaling pathways. Most of the DEGSs tended to be downregulated during callus formation from *P. ternata* hydroponic cuttings, while a few showed an upregulation trend.

The auxin signaling network regulation during callus differentiation is very complex. One hundred four DEGs were involved in the auxin signal transduction pathway, accounting for the largest proportion of all hormone-related DEGs. Auxin-related DEGs involved four *AUX1* [*auxin influx vector* (*AUX1 LAX* series)], three *TIR1* (*transport inhibitor response 1*), 37 *AUX/IAA* (*auxin-responsive protein IAA*), 33 *ARF* (*auxin-responsive factor*), 10 *CH3* (*auxin-responsive CH3* gene family), and 17 *SAUR* (*SAUR family protein*), and so forth (**Figure 4A**). Among them, *AUX1*, *AUX/IAA*, and *ARF* DEGs mainly showed a decreasing expression trend, while an increasing expression trend was observed with *TIR1*, *CH3*, and *SAUR* DEGs. Notably, four *CH3* genes and eight ARF genes were significantly expressed only at 8d, suggesting that these two genes may be key regulators for the initiation of the callus regeneration system. The *AUX/IAA* genes (Cluster-51000.4), *TIR1* gene (Cluster106749.3), and *SAUR* gene (Cluster- 57862.0) were significantly upregulated only at 30d, which may be a key regulatory gene for shoot differentiation after callus formation. Further experimental studies are needed to confirm the function of these genes.

A total of 33 DEGs were involved in the CK signaling transduction pathway, and the related DEGs included 11 *CRE1* [*Arabidopsis histidine kinase 2/3/4* (*cytokinin receptor*)], one *AHP* (*histidine-containing phosphotransfer protease*), 12 *B-ARR* (*two-component response regulator ARR-B series*), eight *A-ARR* (*two-component response regulator ARR-A series*), and others (**Figure 4B**). Most of these genes were activated at 15d callus stage, including two *CRE1* genes, one *B-ARR* gene, and six *A-ARR* genes. CK rapidly activates the expression of A-type *ARR*s, a family of genes considered to be negative feedback inhibitors of the CK response. It is worth emphasizing that the expression of *AHP* was significantly upregulated at 8d but was then downregulated. *B-type ARR* can promote the transduction of CK response. The gene Cluster-49725.0 of *B-ARR* was strongly activated only at 8d, while Cluster-68036.0 was significantly activated only at 30d.

A total of 36 DEGs were involved in the ABA signaling pathway. The related DEGs included seven *PYR/PYL* (*abscisic acid receptor PYR/PYL family*), 11 *PP2C* (*protein phosphatase 2C*), eight *SNrk2* (*serine/threonine protein kinase SRK2*), and eight *ABF* (*ABA response element binding factor*) (**Figure 4C**). Among them, two *SNrk2* genes and two *PP2C* genes were significantly upregulated at 25d. Most of the *PYR/PYL* family genes were significantly upregulated at 30d. Only one *ABF* family gene (Cluster-98067.3) showed q higher expression at 15d.

A total of 42 DEGs were involved in the JA signaling pathway, and the related DEGs involved seven *JAR1* (*jasmonic acid-amino synthetase*), seven *COI1* (*jasmonic acid signaling receptor* and *coronin insensitive protein 1*), 19 *JAZ* (*jasmonate ZIM structural domain protein*), seven *MYC2* (*transcription factor MYC2*), and others (**Figure 4D**). Most genes exhibited their highest expression at 0d and showed a gradually decreasing trend. Among the *JAR1* family genes, Cluster-70814.8 was significantly upregulated at 30d, the *JAZ* gene Cluster-45192.15 was most significantly expressed at 8d, and Cluster-32815.11 was most abundantly expressed at 30d.

#### 3.4.3 Analysis of key differentially expressed genes involved in plant hormone biosynthesis pathways

Gene-level analysis of plant hormone metabolic pathways revealed 487 shared DEGs associated with hormone synthesis pathways during callus development of *P. ternata* in hydroponically grown cuttings of *P. ternata*. These included DEGs involved in the auxin, CK, JA, and ABA biosynthesis pathways.

**Figure 5:**
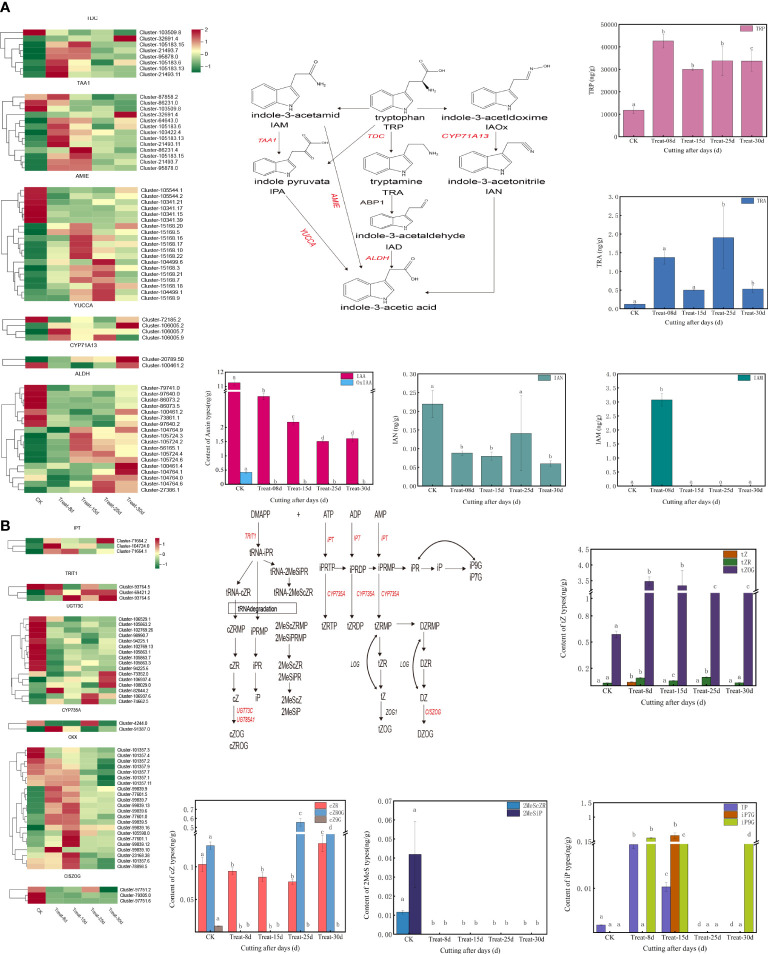
**(A)** Changes in expression of genes and metabolites of the auxin biosynthesis pathway. **(B)** Changes in the expression of genes and metabolites of the cytokinin biosynthetic pathway.

A total of 126 DEGs were involved in the tryptophan metabolic pathway of auxin biosynthesis, accounting for the largest proportion of all hormone synthesis-related DEGs. The auxin-related DEGs included *TDC* (*L-tryptophan decarboxylase*), *TAA1* (*L-tryptophan–pyruvate aminotransferase*), *YUCCA* (*indole-3-pyruvate monooxygenase*), *ALDH* [*aldehyde dehydrogenase (NAD+*)], *AMIE* (*amidase*), and others (**Figure 5A**). Among them, three *TDC* genes, four *TAA1* genes, and one *YUCCA* gene were significantly upregulated at the 8d callus stage. One *TDC* gene, two *TAA1* genes, and four *AMIE* genes were significantly expressed at 15d compared with 0d. Five *AMIE* genes, one *YUCCA* gene, and two *ALDH* genes were highly upregulated at the 25d callus stage.one *TDC* gene, one *TAA1* gene, one *YUCCA* gene, and two *ALDH* genes were highly upregulated at 30d.

A total of 84 DEGs are involved in the CK biosynthetic pathway, with DEGs including *IPT* [*adenylate dimethylallyl transferase* (*cytokinin synthase*)], *CYP735A* (*cytokinin trans-hydroxylase*), *TRIT1* (*tRNA dimethylallyltransferas*), *UGT73C* (*UDP-glucosyltransferase 73C*), *CKX* (*cytokinin dehydrogenase*), *CISZOG* (*cis-zeatin O-glucosyltransferase*), and others (**Figure 5B**). Among them, one IPT gene, one *TRIT1* gene, one *UGT73C* gene, one *CYP735A* gene, and one *CKX* gene were significantly upregulated at 8d callus stage. One *TRIT1* gene and six *CKX* genes were significantly activated at 15d. Two *UGT73C* genes and one *CYP735A* gene were significantly upregulated at 25d. One *IPT* gene, two *TRIT1* gene, and three *UGT73C* genes were significantly induced at 30d. One *IPT* gene, two *TRIT1* genes, and three *UGT73C* genes were significantly upregulated at 30d. Notably, the *TRIT1* gene (Cluster-69421.2) remained highly expressed at 25d and 30d.

**Figure 6:**
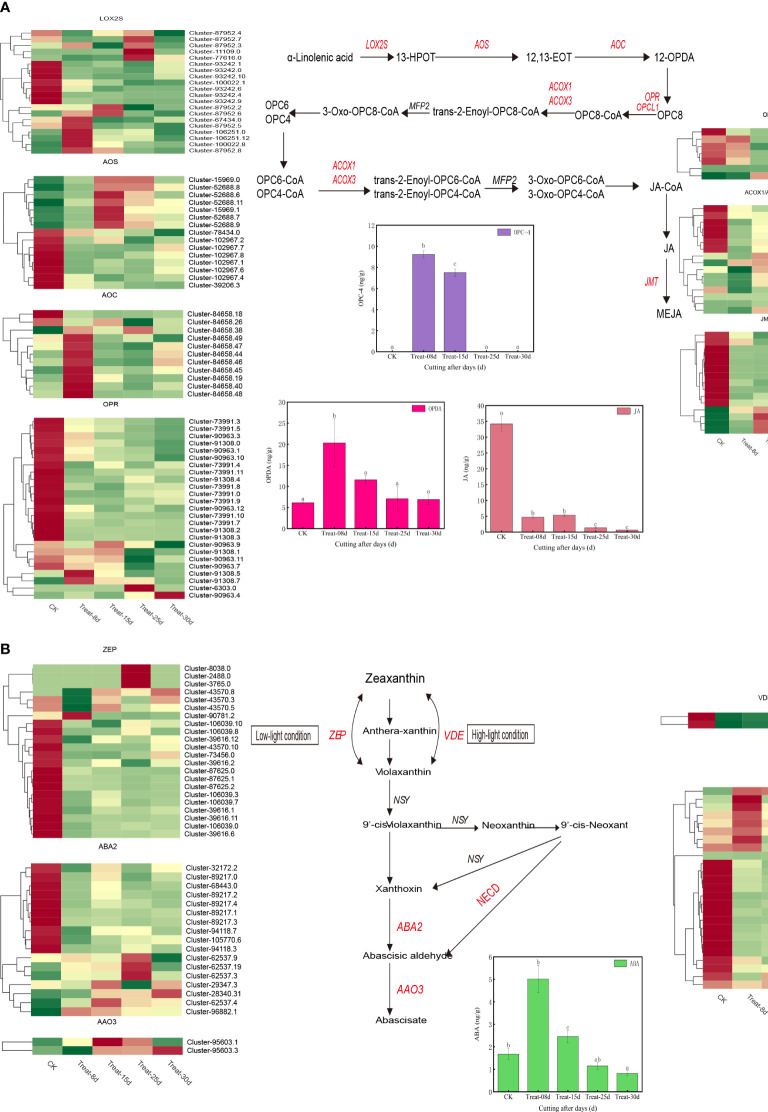
**(A)** Changes in expression of genes and metabolites of the auxin biosynthesis pathway. **(B)** Changes in the expression of genes and metabolites of the cytokinin biosynthetic pathway.

The jasmonic acid synthesis pathway involved in 109 DEGs. They included LOX2S (lipoxygenase), AOS (hydroperoxide dehydratase), AOC (allene oxide cyclase), OPR (12-oxophytodienoic acid reductase), OPCL1 (OPC-8:0 CoA ligase 1), ACOX1/ACOX3 (acyl-CoA oxidase), JMT (jasmonate O-methyltransferase), and others (**Figure 6A**). The expression of six LOX2S genes, eight AOC genes, and two OPR genes were significantly upregulated at 8d. Four AOS genes were significantly upregulated at 15d. Three LOX2S genes, one OPR gene, and four ACOX1/ACOX3 genes were significantly upregulated at 25d. One OPR gene and one OPCL1 gene were significantly increased at 30d. Three LOX2S genes, one OPR gene, and four ACOX1/ACOX3 genes were significantly upregulated at 25d.

Finally, 68 DEGs were involved in the ABA biosynthetic pathway and included the *ZEP/ABA1* (*zeaxanthin epoxidase*), *VDE* (*violaxanthin de-epoxidase*), *NECD* (*9-cis-epoxycarotenoid dioxygenase*) *ABA2* (*xanthoxin dehydrogenase*), *AAO3* (*abscisic-aldehyde oxidase*), and others (**Figure 6B**). Among them, one *ZEP* gene, three *NECD* genes were significantly activated at 8d. One AAO3 gene was significantly upregulated at 15d. Three *ZEP* genes, one *NECD* gene, two *ABA2* genes were significantly induced at 25d and one *AAO3* gene was significantly induced at 30d.

#### 3.4.4 Analysis of transcription factors during callus formation

The TF (transcription factor) expression levels were further analyzed during the callus formation process, as TF-mediated regulation is crucial for all stages of callus development. We identified 2,659 TF genes from all DEGs. Based on the TF gene expression levels, nine TF families with high expression were further analyzed, mainly including *bHLH* (*basic helix-loop-helix basic helix-loop-helix*, 169 members), *AP2/ERF-ERF* (*ethylene response factor*, 190 members), *C2H2* (*cysteine-2/histidine-2*, 90members), *WARK* (*WRKY transcription factor*, 141 members), *CH3* (54 members), *NAC* (87 members), *bZIP* (59 members), *MYB-related* (*myeloblastosis related*, 81 members), and *AUX/IAA* (*auxin-responsive protein IAA*, 102 members).

**Figure 7:**
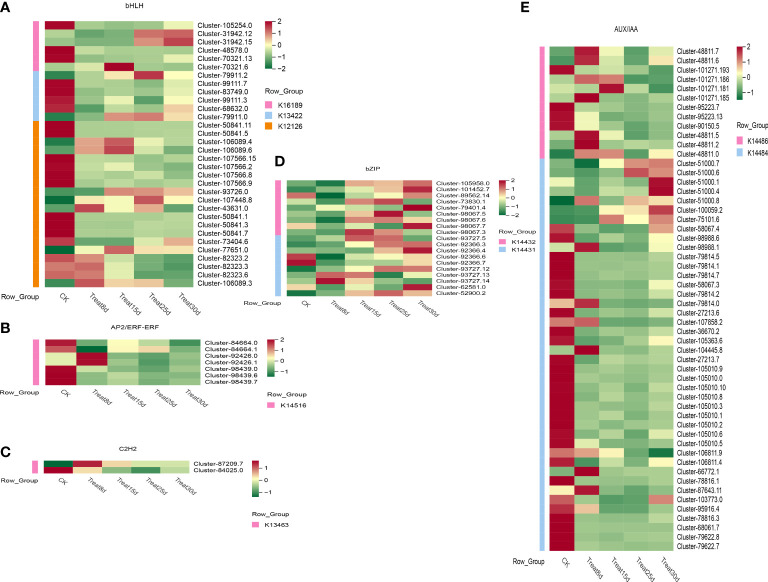
Changes in gene expression in phytohormone signaling in developing callus. **(A)** Expression levels of the *bHLH* TFs family differentially expressed genes (DEGs). **(B)** Expression levels of the *AP2/ERF-ERF* TFs family DEGs. **(C)** Expression levels of the *C2H2* TFs family DEGs. **(D)** Expression levels of the *bZIP* TFs family DEGs. **(E)** Expression levels of the *AUX/IAA* TFs family DEGs.

We identified 67 *bHLH* transcription factors genes during the callus formation (**Figure 7A**), including K16189-*photosensitive pigment signaling interactions factor* (*PIF4*), K13422-transcriptional factor *MYC2*, K1212-transcriptional factor *MYC2*, K1212-*transcriptional protein IAA*, and K1212-*transcriptional protein IAA factor MYC2*, K12126-*photosensitive pigment interaction factor 3* (*PIF3*), and others. Only one *PIF4* was strongly expressed at 15d. The *MYC2* TF (Cluster-79911.2) was strongly activated at 25d. Among *AP2/ERF-ERF*, seven genes were identified (**Figure 7B**), of which two TFs were strongly activated at 8d, especially the K14516-*ethylene response transcription factor 1* (*ERF1*). In *C2H2* TF family (**Figure 7C**), two genes were found to be associated with K13463-*coronin insensitive protein 1* (*COI1*). Nineteen *bZIP* genes were found (**Figure 7D**), including the K14432-*ABA response element binding factor* (*ABF*) and K14431-*transcription factor TGA*. One hundred ten *AUX/IAA* genes were identified (**Figure 7E**), including the K14486-*auxin response factor* (*ARF*), K14484-*auxin-responsive protein IAA* (*IAA*), most of which were significantly expressed in control (CK). Additionally, four *ARF* TFs were significantly highly expressed at 8d, and only one was significantly upregulated at 15d. Moreover, four *IAA* TFs were upregulated at 8d, and two TFs were significantly upregulated at 30d.

### 3.5 Quantitative polymerase chain reaction expression analysis of genes involved in plant hormone biosynthesis and signaling pathways

To experimentally confirm the expression of the unigenes identified by sequencing and computational analysis, seven representative DEGs related to plant hormone synthesis and signaling pathways including *AUX/IAA*, *ARF*, *A-ARR*, *COI1*, *JAZ*, *AOC*, and *OPCL1* were selected for qRT-PCR analysis across the five sequential developmental stages in *P. ternata* hydroponic cuttings.

Based on the analyzed qRT-PCR data (**Supplementary Figure S7**; **Supplementary Table S9**), these DEGs were expressed at varying levels in different stages. Moreover, the qRT-PCR results were generally consistent with the RNA-seq data, indicating transcriptome analysis the reliability. These results also suggest that the evaluated genes potentially play important roles in plant hormone synthesis, signal transduction and distribution, which lead to callus induction.

## 4 Discussion

**Figure 8:**
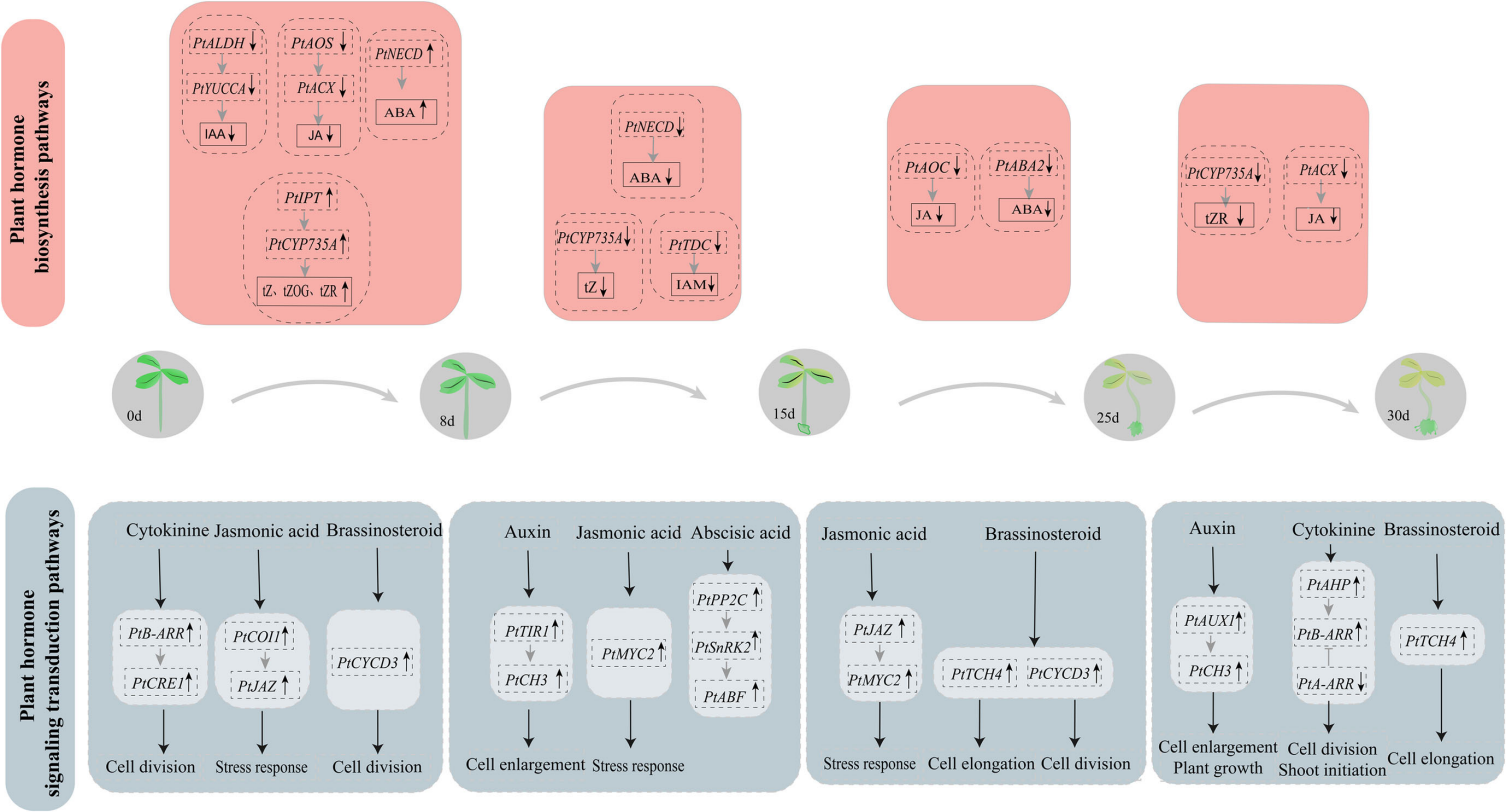
Proposed model of callus proliferation regulation in *P. ternata* hydroponic cuttings.

Our group has developed the first efficient cultivation technique of “hydroponic cuttings from *P. ternata*” using liquid as the cultivation medium. Callus induction is the key link of this technique, but the mechanism of callus formation and development has thus far remained unclear. Therefore, in order to further investigate the molecular mechanisms of callus formation and development in hydroponic cuttings, we provided *P. ternata* hormone metabolism and transcriptome data. These data included the hormone metabolic compounds and DEGs involved in hormone metabolism and signal transduction pathways of the callus in the hydroponic cuttings at different stages (**Figure 8**).

The process of callus development is closely linked to its endogenous hormone homeostasis and dynamic changes. We found that CKs, auxin, JA, and ABA-type derivatives are present mainly in biologically active form throughout callus development from hydroponic cuttings (**Figures 2**, **3**; **Supplementary Figures S3**, **S4**). This confirms that callus development is a highly dynamic process under hormonal control (Perez-Garcia and Moreno-Risueno, 2018). All hormones reached high concentrations throughout callus development, with a major peak occurring at 15d and a lower peak in concentration at 8d (**Supplementary Figure S8**). This also indicates again that endogenous hormones play an indispensable role in the early stages of callus formation (Perez-Garcia and Moreno-Risueno, 2018).

In previous studies, auxin was shown to play an important role in tissue culture, promoting callus formation and adventitious root formation in cuttings (Che et al., 2002). The content of IAA increased 1–3 days after the flowering stems of Arabidopsis were cut (Cui et al., 2021). However, in this study, we showed that auxin content was regulated by *ALDH*, *YUCCA*, and *TAA1*, gradually decrease during callus formation and during callus proliferation, indicating that low auxin concentrations were beneficial to callus formation as well as its development. In terms of auxin signal activation, three major primary response gene families were induced: *AUX/IAA* genes, *GH3* genes (Hayashi et al., 2021), and *SAUR* genes (Che et al., 2002). The *AUX/IAA* family members are short-lived nuclear proteins that form homo- and heterodimers with other *AUX/IAA* and *ARF* genes and repress transcription of downstream auxin response genes (Chen et al., 2019). Eight *AUX/IAA* genes were differentially expressed in *P. ternata* cuttings during callus formation, and only one was significantly expressed at 30d. The *SAUR* family is the largest known family of early auxin-inducible genes (Ren and Gray, 2015), and *OsSAUR39* has been reported to regulate auxin biosynthesis and transport negatively. Interestingly, one *SAUR* gene was differentially expressed at 30d late in *P. ternata* callus formation, suggesting that this gene facilitates late callus differentiation to form shoots. Therefore, their role in this process needs further investigation. Several auxin biosynthetic genes regulated the decreasing IAA concentration at the gene expression level. Overall, the low level of IAA is beneficial to the formation of calli.

CKs are essential in grafted plants, especially in callus formation and vascular system development. Notably, CK responses were elevated in Arabidopsis explants (Iwase et al., 2011). In contrast, CKs regulate plant growth and development through a two-component regulatory system (Che et al., 2002). CKs were found to control the shoot meristem development by stimulating the upregulation of *A-ARR* genes and suppressing *WUS* expression. In addition, *CRE1*, *AHP*, and *B-ARR* act as upstream regulators of *A-ARR* (Buechel et al., 2010). One *CRE1* gene and five *A-ARR* genes were significantly upregulated during *P. ternata* callus induction, suggesting they play an important role in CK signaling and callus formation. The hormone metabolism levels gradually increased for most of the CKs and were regulated by several biosynthetic pathway genes, such as *IPT*, *CYP735A*, and *CKX*. However, cZROG, which may be regulated by the *UGT73C* gene, decreased during the early callus formation stage and then increased at the differentiation stage of shoot formation. Moreover, cZROG may be a key factor in shoot formation. In conclusion, CK is the core hormone controlling the formation of calli in *P. ternata* hydroponic cuttings.

JA is well-known as a stress-induced hormone. Previous studies found that JA prevents plant growth (Yan et al., 2007) and inhibits cell proliferation and expansion to counter-regulate organ development (Noir et al., 2013). In the present study, three *JAZ* genes were significantly upregulated. The level of JA inhibition was low during the process of *P. ternata* callus formation, indicating that low concentrations of JA can promote rapid callus proliferation and development, consistent with the results of garlic callus proliferation studies (Mostafa et al., 2020). ABA negatively regulates plant growth by inhibiting cell proliferation under stress conditions (Wang et al., 1998). The ABA levels, and key genes for ABA synthesis, were regulated at 8d, suggesting ABA plays an important role in the rapid *P. ternata* callus proliferation, contrary to previous studies.

Plant endogenous hormones form a complex signaling network that affects cell growth and development by regulating key genes for cell proliferation (Ahuja, 1965; Hofhuis et al., 2013). Plants can respond rapidly to external stimuli, leading to changes in gene transcriptional levels. Moreover, a large number of transcription factors are responsive to external stresses. We identified a differential expression of transcription factors such as *PIF*, *MYC*, *ERF*, *TGA*, and *ARF* was found, suggesting they may be involved in gene network regulation during *P. ternata* callus formation from hydroponic cuttings. *PIF4* can positively regulate elongation growth in plants by regulating the expression of auxin genes encoding, affecting downstream signal transduction pathways (Sun et al., 2013). In the present study, early callus grew faster, and the expression of *PIF* TFs was suppressed, resulting in decrease IAA content. *ARF* transcription factors are key components in the auxin signaling pathway and repress or activate the expression of downstream genes by binding to upstream response elements of auxin response genes. Eight *ARF* genes were significantly upregulated in the callus at the 8d stage, suggesting their potential involvement in callus induction and development. *MYC* transcription factors are JA-related gene expression activators or repressors that regulate plant growth and development (Aleman et al., 2016). *ERF* transcription factors occur in plants and are involved in plant responses to biotic and abiotic stresses (Ding et al., 2021). In *Cercis canadensis*, taxol biosynthesis is regulated by ERF transcription factors and is dependent on JA signaling. It is possible that the expression of *MYC* TFs was repressed, resulting in reduced JA content. *TGA* TFs are members of the *bZIP* family and play an important role in developing stress tolerance (Dröge-Laser et al., 2018; Wang et al., 2020; Ke et al., 2022). In Arabidopsis, *TGA* TFs enhance resistance by regulating the auxin signaling (Choi et al., 2010). In this study, *TGA* expression may be maintained at low levels to promote callus development.

## 5 Conclusion

In conclusion, our study evaluated the endogenous hormone content and performed transcriptome sequencing at different stages of callus of *P. ternata* hydroponic cuttings. Genes co-expressed in phytohormone synthesis and transduction were analyzed at each stage and were combined with hormone metabolite data and transcriptomics to reveal the molecular mechanism of callus formation from *P. ternata* hydroponic cuttings. CKs were the hormones with the highest concentration during the whole callus development. In the early stage of callus formation (8d), CK levels were gradually increased through the regulation of *IPT* and *CYP735A* genes. AHP positively regulated B-ARRs and CRE1, promoting CK signaling and facilitating callus cell differentiation. The brassinosteroid signaling gene Cyclin D3 (CYCD3) was upregulated, facilitating the initiation of callus cell differentiation. The *B-ARR* gene promoted cell differentiation and thus induced callus formation through regulation of the *ALDH* and *YUCCA* genes, which reduced the IAA levels, facilitating the formation and development of callus. *AOS*, *ACX* negatively regulated JA synthesis, reducing JA levels, and promoting callus formation in the cutting wound site. Moreover, COI1 and JAZ positively regulated JA signaling and promoted plant stress and wound responses at the wound site. During the middle and late stages of callus formation (15d, 25d, and 30d), the endogenous hormone content in the callus was decreasing. However, specific hormone signaling functions positively regulated the formation and development of the callus. At 15d, the auxin signaling genes *TIR1* and *CH3* were upregulated, promoting callus cell proliferation. The upregulation of *MYC* transcription factors involved in JA signaling also indicated that JA regulated the response to mechanical damage of *P.ternata* hydroponic cuttings. At 25d, the brassinosteroid signaling genes *TCH4* and *CYCD3* were upregulated, promoting the differentiation and proliferation of callus cells. At 30d, the auxin-signaling genes *AUX1* and *CH3* positively regulated callus development and promoted cell proliferation and plant growth. The CK signaling genes *AHP* and *B-ARR* were upregulated to promote continuous cell differentiation and shoot initiation. Plant endogenous hormones are particularly important for callus formation and development. The results of this study elucidate for the first time the molecular mechanism of the callus formation process in *P. ternata* cuttings grown in hydroponic, providing new insights and approaches for expanding *P. ternata* resources and propagation practices.

## Data availability statement

The datasets presented in this study can be found in online repositories. The names of the repository/repositories and accession number(s) can be found below: https://www.ncbi.nlm.nih.gov/, PRJNA942460.

## Author contributions

XD, HC, FW and YH designed the experiments and contributed to writing and revising the manuscript. XD and LC performed the experiments. XD and LC analyzed the data. YL supervised this study. All authors contributed to this paper and have approved the submission.

## Footnotes

1 UniProtKB/Swiss-Prot - SIB Swiss Institute of Bioinformatics | Expasy, n.d

2
http://www.metware.cn/

3
https://github.com/trinityrnaseq/trinityrnaseq

4
https://www.kegg.jp/kegg/kegg1.html

5
http://www.omicshare.com/tools

6
https://bioconductor.org/packages/DESeq2/, version3.10
