# Supplementary material for: Integrated physicochemical, hormonal, and transcriptomic analysis reveals the underlying mechanism of callus formation in Pinellia ternata hydroponic cuttings
Source: Front Plant Sci. 2023 Jun 20;14:1189499. doi: 10.3389/fpls.2023.1189499 (PMC10319145; doi:10.3389/fpls.2023.1189499)
Supplement: Supplementary file 1 [file DataSheet_1.pdf]

Supplementary Material

1 Supplementary Figures and Tables

1.1 Supplementary Figures

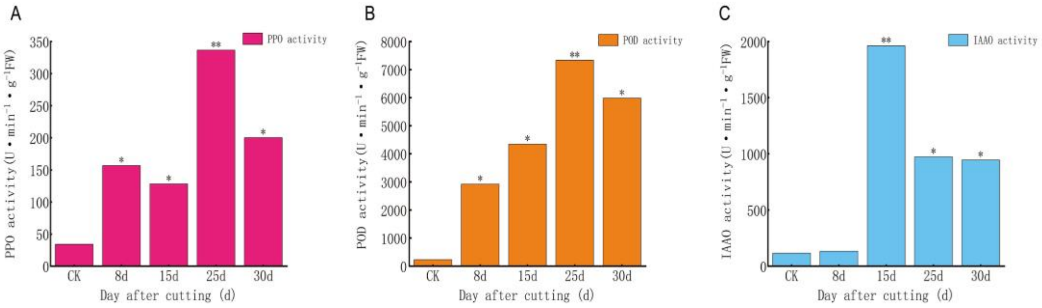

Supplementary Figure 1 Oxidase activities in five stages of *P. ternata* callus formation from hydroponic cuttings. (A) Change in peroxidase activity (POD); (B) change in polyphenol oxidase activity (PPO); (C) changes in indoleacetic acid oxidase (IAAO) activity. The error bars represent the SD from three biological replicates. *, statistical significance at $P < 0.05$; **, statistical significance at $P < 0.01$.

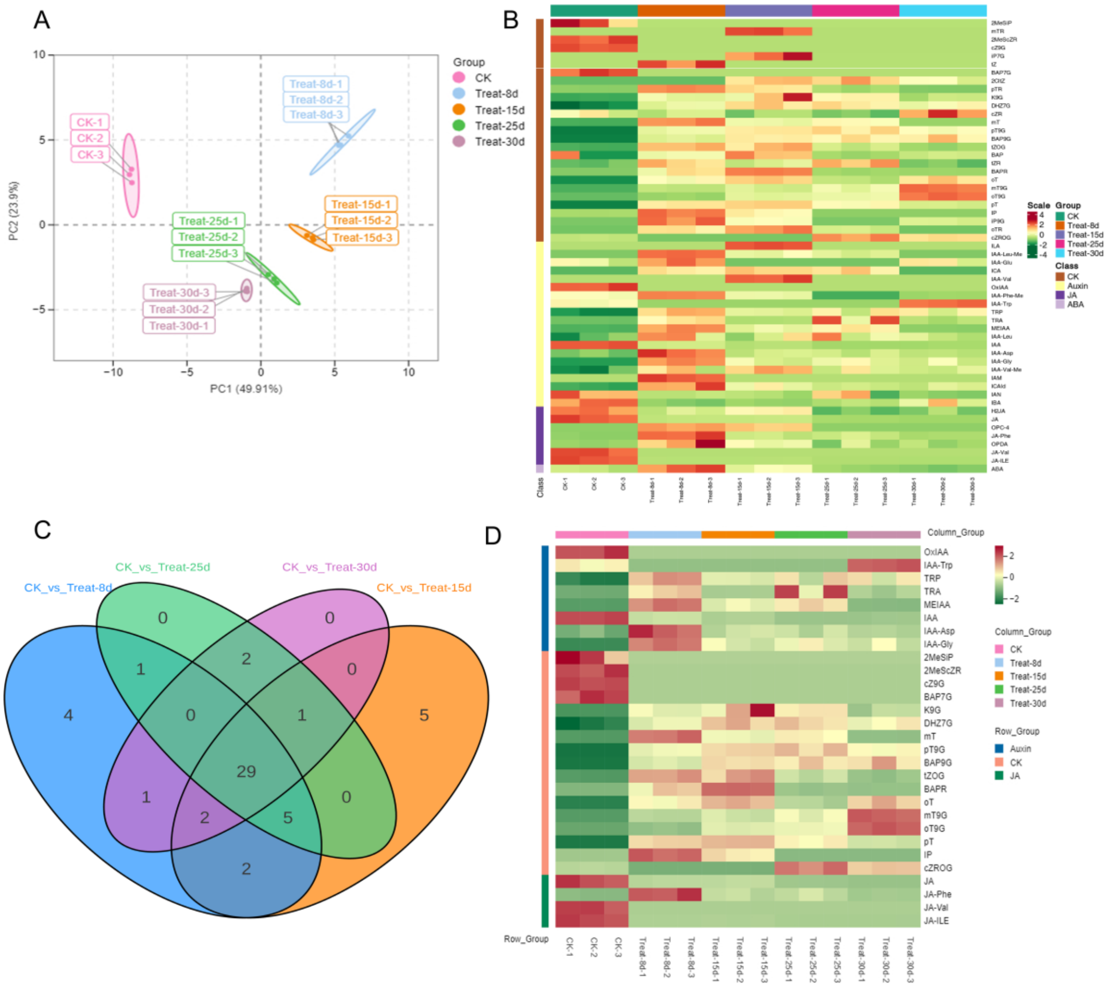

Supplementary Figure 2 (A) PCA score; (B) clustering heat map of all targeted metabolites; (C) Venn diagram of differential metabolites; (D) clustering heat map of 29 differential metabolites.

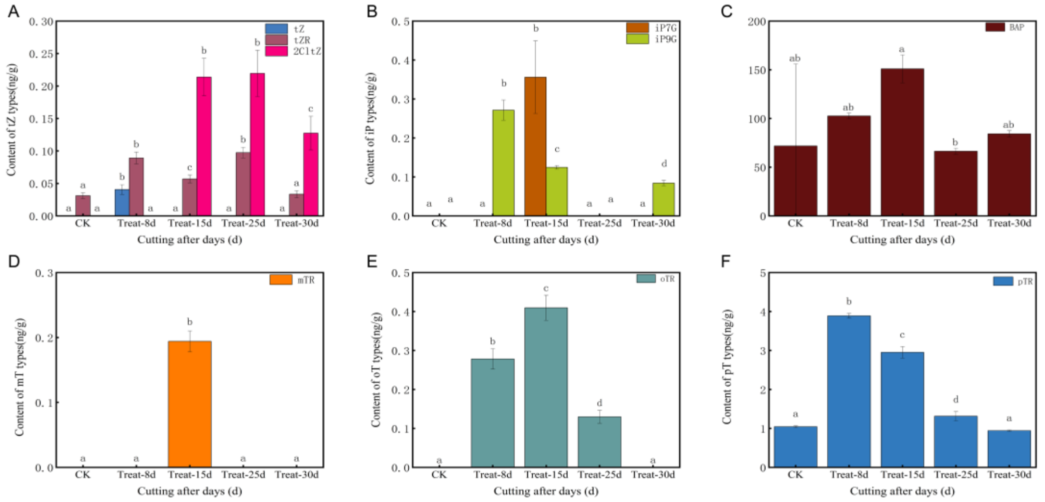

Supplementary Figure 3 Changes in endogenous cytokinins (CKs) content at five stages of *P. ternata* hydroponic cuttings callus formation. (A) trans-zeatin (tZ) type; (B) N6-(Δ2-prenyl) adenine (iP) type; (C) 6- benzylaminopurine (BAP) type; (D) 3-[(9H-purin-6-ylamino) methyl] phenol (mT) type;(E) 4-[(9H-purin-6-ylamino) methyl] phenol (oT) type;(F) 2-[(9H-purin-6-ylamino) methyl] phenol (pT) type. The bars with different letters are significantly different from each treatment ($p < 0.05$). Values are means of three replicates ± SE.

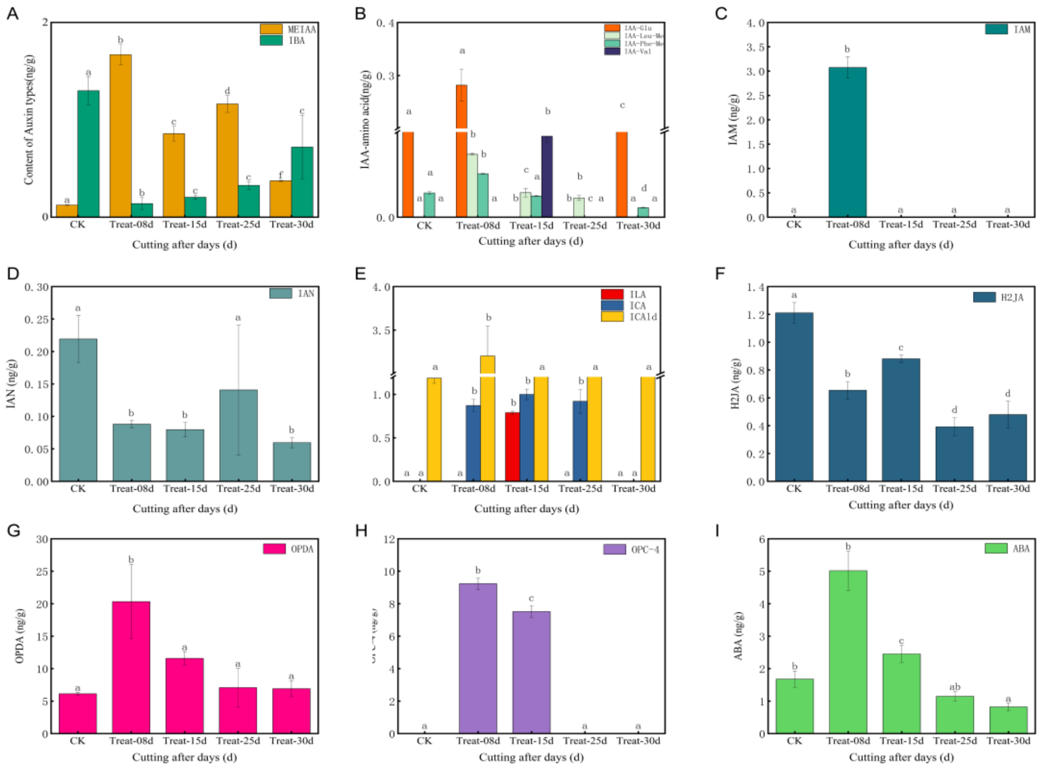

Supplementary Figure 4 Changes in endogenous auxin, jasmonic acid, and abscisic acid contents in five stages of *P. ternata* hydroponic cuttings callus formation (A)IAA active substance;(B) Amino acid conjugates substance of IAA ;(C) IAM precursor substance;(D) IAN precursor substance; (E) IAA precursor substances (F)H2JA;(G) JA precursor substances; (H) JA precursor substances; (I) ABA. The bars with different letters are significantly different from each treatment ($p < 0.05$). Values are means of three replicates ± SE.

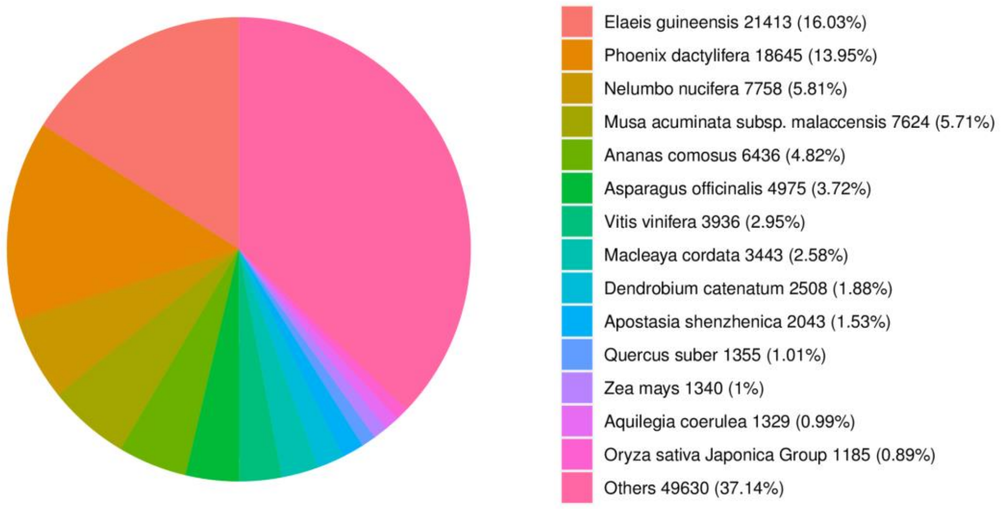

Supplementary Figure 5 Unigene species identified during callus development.

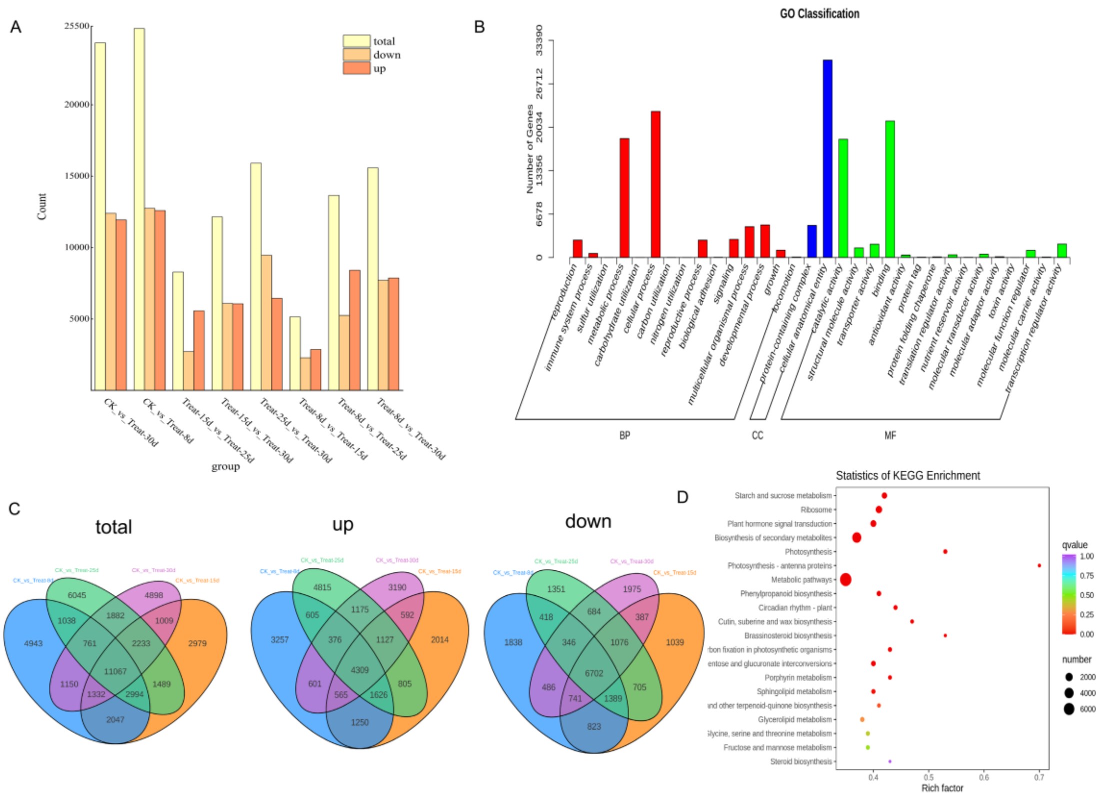

Supplementary Figure 6 Functional annotation and classification of genes involved in *P. ternata* callus induction from hydroponic cuttings. (A) The number of up- and down-regulated DEGs at different stages of *P. ternata* callus induction; (B) GO classification of DEGs in *P. ternata* callus induction; (C) Venn diagram of annotated texts from multiple databases; (D) KEGG enrichment analysis of DEGs in *P. ternata* callus induction.

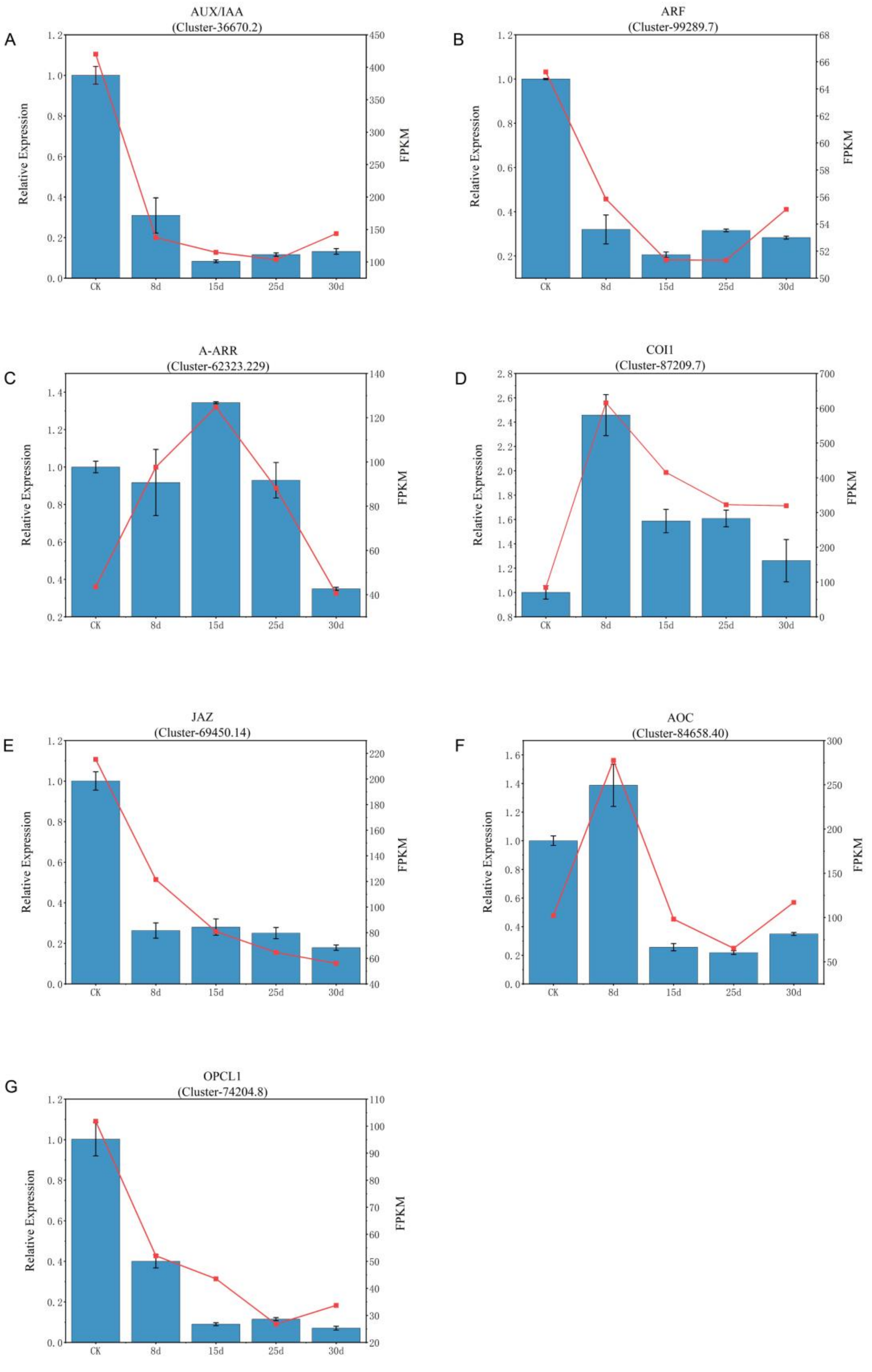

Supplementary Figure 7 Validation of the expression of Plant hormone synthesis pathways and signaling Genes during *P. ternata* callus formation from hydroponic cuttings. The expression level of 7 callus development-related genes in different stages

of *P. ternata* callus formation was validated by qRT-PCR. The data were based on the analysis of three independent biological repeats.

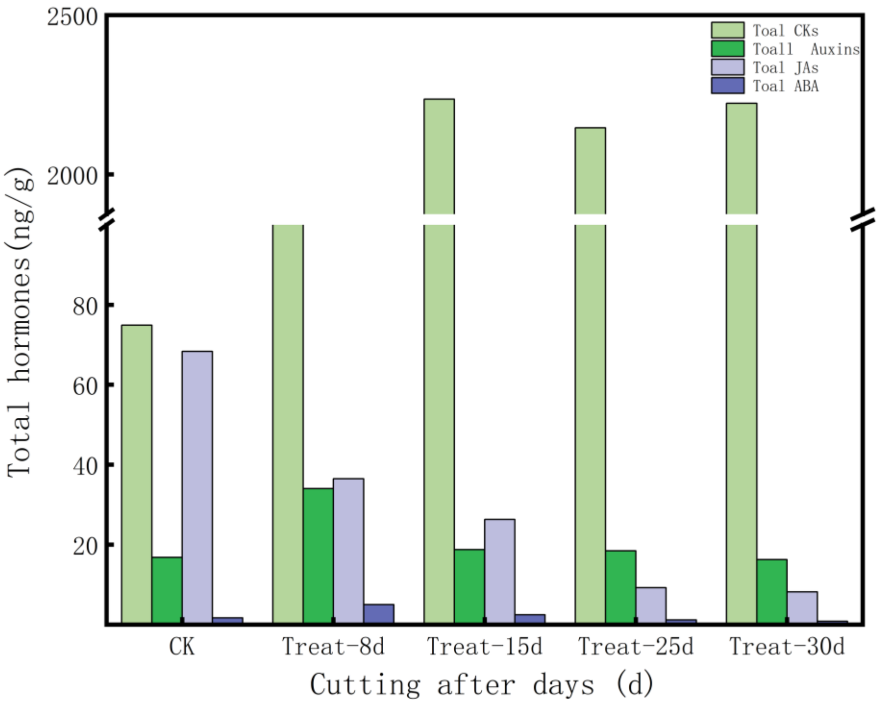

Supplementary Figure 8 The sum of various hormones during the formation of callus in hydroponic cuttings.

1.2 Supplementary Tables

Supplementary Table 1 29 shared differential metabolites in the four comparison groups.

Index	Compounds	Class
TRP	L-tryptophan	Auxin
TRA	Tryptamine	Auxin
IAA	Indole-3-acetic acid	Auxin
OxIAA	2-oxindole-3-acetic acid	Auxin
MEIAA	Methyl indole-3-acetate	Auxin
IAA-Trp	Indole-3-acetyl-L-tryptophan	Auxin
IAA-Asp	Indole-3-acetyl-L-aspartic acid	Auxin
IAA-Gly	Indole-3-acetyl glycine	Auxin
2MeSiP	2-Methylthio-N6-isopentenyladenine	CKs
2MeScZR	2-Methylthio-cis-zeatin riboside	CKs
tZOG	trans-Zeatin-O-glucoside	CKs
cZ9G	cis-Zeatin-9-glucoside	CKs
cZROG	cis-Zeatin-O-glucoside riboside	CKs
IP	N6-isopentenyladenine	CKs
DHZ7G	Dihydrozeatin-7-glucoside	CKs
BAPR	6-Benzyladenosine	CKs
BAP7G	N6-Benzyladenine-7-glucoside	CKs
BAP9G	N6-Benzyladenine-9-glucoside	CKs
K9G	Kinetin-9-glucoside	CKs
mT	meta-Topolin	CKs
mT9G	meta-Topolin-9-glucoside	CKs
pT	para-Topolin	CKs
pT9G	4-[[(9-beta-D-Glucopyranosyl-9H-purin-6-yl)amino]methyl]phenol	CKs
oT	ortho-Topolin	CKs
oT9G	ortho-Topolin-9-glucoside	CKs
JA	Jasmonic acid	JA
JA-ILE	Jasmonoyl-L-isoleucine	JA
JA-Phe	N-[(-)-Jasmonoyl]-(l)-phenalanine	JA
JA-Val	N-[(-)-Jasmonoyl]-(L)-valine	JA

Supplementary Table 2 The RNA sequencing quality of callus development in *P. ternata*.

Sample	Raw Reads	Clean Reads	Clean Base(G)	Q20(%)	Q30(%)	GC content (%)
CK-1	51524586	49467500	7.42	97.78	94.03	54.82
CK-2	58906622	56747580	8.51	97.71	93.79	54.54
CK-3	54569012	52390030	7.86	97.76	93.93	54.46
Treat-8d-1	71285616	68581176	10.29	97.6	93.59	53.94
Treat-8d-2	54411186	51564830	7.73	97.26	93.19	53.81
Treat-8d-3	58784490	56848366	8.53	97.84	94.11	53.24
Treat-15d-1	41276676	39297936	5.89	97.27	92.83	53.28
Treat-15d-2	60379320	58212710	8.73	97.66	93.74	53.75
Treat-15d-3	64699366	61628900	9.24	97.69	93.77	53.18
Treat-25d-1	70507230	68012578	10.2	97.56	93.44	53.4
Treat-25d-2	43805062	42079016	6.31	96.64	91.62	53.34
Treat-25d-3	54603428	52524838	7.88	97.53	93.31	53.63
Treat-30d-1	58076330	55688242	8.35	97.71	93.6	53.54
Treat-30d-2	58403302	56044258	8.41	97.61	93.49	53.27
Treat-30d-3	44263598	42632560	6.39	97.32	92.84	53.35

Supplementary Table 3 Gene functional annotation for callus development.

Database	Number of Genes	Percentage (%)
KEGG	98381	38.71
Nr	132435	52.11
SwissProt	92351	36.34
TrEMBL	137465	54.09
KOG	78766	30.99
GO	111436	43.85
Pfam	91057	35.83
Annotated in at least one Database	144179	56.73
Total Unigenes	254137	100

Supplementary Table 4 The numbers of DEGs in CK vs. 8d, CK vs. 15d d, CK vs. 25d, and CK vs. 30d callus stages.

Comprised group	total	down	up
CK_vs_Treat-8d	25332	12743	12589
CK_vs_Treat-15d	25150	12862	12288
CK_vs_Treat-25d	27509	12671	14838
CK_vs_Treat-30d	24332	12397	11935

Supplementary Table 5 KEGG pathway enrichment analysis of DEGs for CK vs the 8d callus stage.

Pathway_ID	Pathway	DEG_number	P-value	Count_up
ko01100	Metabolic pathways	3978	0	1985/1993
ko01110	Biosynthesis of secondary metabolites	2181	0	1074/1107
ko04626	Plant-pathogen interaction	596	0.035834	215/381
ko04075	Plant hormone signal transduction	521	2.08E-08	206/305
ko01200	Carbon metabolism	444	0.000177	201/243
ko00500	Starch and sucrose metabolism	426	0	278/148
ko04016	MAPK signaling pathway - plant	324	0.010922	156/168
ko00040	Pentose and glucuronate interconversions	252	1.89E-09	171/81
ko00010	Glycolysis / Gluconeogenesis	240	1.83E-05	139/101
ko00940	Phenylpropanoid biosynthesis	233	5.21E-11	151/82
ko00195	Photosynthesis	180	4.39E-12	8/172
ko00561	Glycerolipid metabolism	173	0.000213	67/106
ko00630	Glyoxylate and dicarboxylate metabolism	171	0.005661	51/120

Supplementary Table 6 KEGG pathway enrichment analysis of DEGs for CK vs the 15d callus stage.

Pathway_ID	Pathway	DEG_number	P-value	Count_up/down
ko01100	Metabolic pathways	3861	6.95703E-44	1864/1997
ko01110	Biosynthesis of secondary metabolites	2145	0	1054/1091
ko04626	Plant-pathogen interaction	595	0.001718298	219/376
ko04075	Plant hormone signal transduction	558	0	228/330
ko00500	Starch and sucrose metabolism	427	0	290/137
ko01200	Carbon metabolism	393	0.047995115	157/236
ko04016	MAPK signaling pathway - plant	340	1.70803E-05	178/163
ko00940	Phenylpropanoid biosynthesis	253	8.87279E-12	180/73
ko00040	Pentose and glucuronate interconversions	230	5.62E-07	133/97
ko00010	Glycolysis / Gluconeogenesis	213	0.003295316	115/98
ko00195	Photosynthesis	166	2.28351E-12	7/159
ko00630	Glyoxylate and dicarboxylate metabolism	165	0.004879277	44/121
ko00561	Glycerolipid metabolism	161	0.001290396	69/92

Supplementary Table 7 KEGG pathway enrichment analysis of DEGs for CK vs the 25d callus stage.

Pathway_ID	Pathway	DEG_number	P-value	Count_up/down
ko01100	Metabolic pathways	4103	0	2192/1911
ko01110	Biosynthesis of secondary metabolites	2246	0	1213/1033
ko03010	Ribosome	909	0	866/43
ko04075	Plant hormone signal transduction	557	4.88E-08	203/354
ko01200	Carbon metabolism	482	8.71E-05	255/227
ko00500	Starch and sucrose metabolism	408	3.53E-09	259/149
ko04016	MAPK signaling pathway - plant	360	0.001761	185/175
ko00940	Phenylpropanoid biosynthesis	242	1.39E-09	171/71
ko00010	Glycolysis / Gluconeogenesis	242	0.001511	150/92
ko00040	Pentose and glucuronate interconversions	228	0.002767	116/112
ko00630	Glyoxylate and dicarboxylate metabolism	191	0.000905	75/116
ko04145	Phagosome	174	0.008514	135/39

Supplementary Table 8 KEGG pathway enrichment analysis of DEGs for CK vs the 30d callus stage.

Pathway_ID	Pathway	DEG_number	P-value	Count_up/down
ko01100	Metabolic pathways	3558	0	1773/1785
ko01110	Biosynthesis of secondary metabolites	2013	0	1024/989
ko04626	Plant-pathogen interaction	575	0.000526	181/394
ko04075	Plant hormone signal transduction	551	0	211/340
ko01200	Carbon metabolism	395	0.002225	174/221
ko00500	Starch and sucrose metabolism	393	0	261/132
ko04016	MAPK signaling pathway - plant	319	9.35E-05	160/159
ko00010	Glycolysis / Gluconeogenesis	215	0.000146	123/92
ko00040	Pentose and glucuronate interconversions	211	2.05E-05	112/99
ko00940	Phenylpropanoid biosynthesis	194	2.29E-06	126/68
ko00630	Glyoxylate and dicarboxylate metabolism	156	0.007754	43/113
ko00561	Glycerolipid metabolism	150	0.004268	88/62

Supplementary Table 9 Primer sequences and annealing temperatures used for qRT-PCR.

Gene Symbol	**Primer**		**5'--3'**	**Product (bp)**
——	PtGAPDH	F	TGCTGGGAATGATGTTGAATG	——
		R	TTGGCATTGTTGAGGGTTTG	
AUX/IAA	Cluster-36670.2	F	CACCGTCCTTGTCCTCGTAG	
		R	TTCTCCTGCTTCACCATCCG	103
ARF	Cluster-99289.7	F	AGCAGGGAATTGCTTTGGGA	
		R	CGGATCATCACCAACGAGCA	179
A-ARR	Cluster-62323.229	F	GCTGATTCTGGAGGGCACAT	
		R	CCACCGCTCTGATCACTCTC	190
COI1	Cluster-87209.7	F	AAGGTGGGCACATAGCAACT	
		R	CTCGCTCTCCCTCACATTCC	235
JAZ	Cluster-69450.14	F	TCCACACGGACAAGAAACCA	
		R	CGCCGGGTTTGGAATCAGAA	219
AOC	Cluster-84658.40	F	ACCACGGGAACCTGAAGAAG	
		R	GGAGTCCTCGTAGGTGAGGT	104
OPCL1	Cluster-74204.8	F	ACCGCAGTGATGGATTTCGT	
		R	AATATCTTGCCGGAGGCGTT	167
